# Menschen mit geistiger Behinderung (MmgB) in der ambulanten medizinischen Versorgung: Barrieren beim Zugang und im Untersuchungsablauf

**DOI:** 10.1007/s00103-023-03655-x

**Published:** 2023-01-16

**Authors:** Randi Wellkamp, Werner de Cruppé, Susanne Schwalen, Max Geraedts

**Affiliations:** 1 Institut für Gesundheitssystemforschung, Department für Humanmedizin, Fakultät für Gesundheit, Universität Witten/Herdecke, Alfred-Herrhausen-Str. 50, 58448 Witten, Deutschland; 2 Institut für Versorgungsforschung und Klinische Epidemiologie, Fachbereich Medizin, Philipps-Universität Marburg, Marburg, Deutschland; 3 Ärztekammer Nordrhein, Düsseldorf, Deutschland

**Keywords:** Gesundheitsversorgung, Zugang zum Gesundheitswesen, Gesundheitsbedarf, Prävention, Hausärztliche Versorgung, Health care, Access to healthcare, Health need, Disease prevention, Primary healthcare

## Abstract

**Hintergrund und Ziel:**

Menschen mit geistiger Behinderung (MmgB) weisen eine erhöhte Morbidität auf. Ihr Zugang zur Gesundheitsversorgung könnte ein Einflussfaktor sein. In Deutschland existieren hierzu nur wenige Daten. Die vorliegende Arbeit geht der Frage nach, welche Barrieren und förderlichen Aspekte für MmgB bei der Inanspruchnahme der ambulanten Versorgung bestehen. Dabei wird ihre eigene Perspektive berücksichtigt sowie die der begleitenden Angehörigen und der Hausärzt:innen.

**Methoden:**

In dieser Querschnittstudie wurden mittels Fragebögen MmgB in 3 Werkstätten für behinderte Menschen sowie deren Angehörige und Hausärzt:innen befragt. Die Daten wurden deskriptiv ausgewertet und die Antworten der MmgB und der Angehörigen teststatistisch verglichen. Die inhaltliche Gliederung folgt dem Modell nach Cantrell (Erkennen eines Behandlungsbedarfs, Zugang zur Gesundheitsversorgung, Untersuchungsablauf).

**Ergebnisse:**

MmgB teilen Beschwerden ihren Angehörigen mit, die sie oft zu Arztbesuchen begleiten. Barrieren sind eher organisatorischer als räumlicher Art. Die Behandlungssituation ist teilweise durch Ängste, Unruhe oder auch das Nichtzulassen von Untersuchungen erschwert. Schwierig ist es, erfahrene Praxen zu finden. Daher wünschen sich Angehörige Listen mit solchen Praxen und medizinische Versorgungszentren für MmgB. Die Sicht der MmgB und ihrer Angehörigen zeigt kaum Unterschiede. Hausärzt:innen geben den erhöhten Behandlungsaufwand, Wunsch nach Fortbildung und angemessener Vergütung an.

**Diskussion:**

Die Angehörigen spielen in der medizinischen Versorgung von MmgB eine wichtige Rolle. Schwierigkeiten in der Versorgung können aus den spezifischen, erhöhten Anforderungen im Umgang mit MmgB entstehen, die sich organisatorisch äußern. Es bedarf einer aktiven Bereitschaft zur Inklusion.

**Zusatzmaterial online:**

Zusätzliche Informationen sind in der Online-Version dieses Artikels (10.1007/s00103-023-03655-x) enthalten.

## Abstract (en)

**Background and aim:**

People with intellectual disabilities (ID) show an increased morbidity. Their access to healthcare could be a contributing factor, but there is little data on this in Germany. This paper addresses the question of what barriers and facilitators exist in the use of medical outpatient healthcare for people with ID, considering their own perspective and the perspectives of their accompanying relatives and their general practitioners (GPs).

**Methods:**

In this cross-sectional study, people with ID in three sheltered workshops, their relatives and their GPs were interviewed by means of questionnaires. The data were evaluated descriptively, and a statistical comparison of the perspectives of the people with ID and their relatives was performed. The content structure follows Cantrell’s pathway model (identifying need, accessing services and interaction during a consultation).

**Results:**

People with ID communicate complaints to their relatives, who usually accompany them to medical appointments. There are more organisational than spatial barriers. The treatment is sometimes impeded by fears, restlessness or not allowing examinations. It is difficult to find experienced health professionals, which is why a list of such practices and, structurally, medical centres for people with ID would be beneficial. The views of people with ID and their relatives show hardly any differences. GPs cite increased treatment effort, desire for further training and appropriate remuneration.

**Conclusions:**

Relatives play an important role in the medical care of people with ID. Difficulties in care can arise from the specific, more complex requirements in treating people with ID, which present as organisational difficulties but also require an active readiness for inclusion.

## Hintergrund

Menschen mit geistiger Behinderung (MmgB) weisen eine erhöhte Morbidität sowohl hinsichtlich somatischer Erkrankungen als auch psychischer Störungen auf und ihre Lebenserwartung liegt trotz eines Anstiegs in den letzten Jahrzehnten weiterhin unter der der Allgemeinbevölkerung [1–8]. Diese Unterschiede stehen im Zusammenhang mit einer gesundheitlichen Ungleichheit, die MmgB erfahren. Johnston gliedert diese in 3 Bereiche: Stigmatisierung mit sozialer Exklusion, Unterrepräsentierung sowie unzureichender Zugang zu einer qualitativ angemessenen Versorgung im Gesundheitssystem [9]. Studien zeigen diagnostische und therapeutische Defizite in der medizinischen Versorgung von MmgB auf, aber auch eine geringere Teilnahme an Prävention und Gesundheitsförderung [1–8, 10].

Mit dem Ziel, diese Ungleichheit abzubauen, wurde die Behindertenrechtskonvention der Vereinten Nationen (UN-BRK) verabschiedet, die seit dem Jahr 2009 mit Ratifizierung im Bundestag geltendes deutsches Recht ist [11]. Sie beförderte im Jahr 2016 die Verabschiedung des Bundesteilhabegesetzes samt Aktionsplan zu dessen Umsetzung und die regelmäßige Veröffentlichung von Teilhabeberichten über die Lebenslagen von Menschen mit Beeinträchtigungen (TB-LMmB; [12, 13]). Im Bemühen, die Exklusion zu verringern, wird angestrebt, MmgB weniger in zentralisierten Großeinrichtungen zu betreuen und sie stattdessen in Hinblick auf Wohnen und Arbeiten mehr in familien- und gemeindenahe Strukturen zu integrieren [1, 4, 14]. Auch die medizinische Versorgung soll nun überwiegend in den ambulanten Strukturen erfolgen; allerdings wurde die notwendige vorbereitende Anpassung und Ausstattung der zahlreichen zuständigen Praxen und Ambulanzen für die spezifischen Erfordernisse der MmgB meist noch zu wenig berücksichtigt [3, 4, 15].

Mehrere Übersichtsarbeiten identifizieren Barrieren und förderliche Faktoren für MmgB beim Zugang und im Verlauf der ambulanten medizinischen Versorgung außerhalb Deutschlands. Alborz [16] weist insbesondere darauf hin, wie schwierig es oftmals ist, Krankheitssymptome bei MmgB wahrzunehmen und zuzuordnen. Sie hebt damit hervor, wie wichtig Angehörige sind, um die medizinische Inanspruchnahme zu initiieren, zu organisieren und auch zu begleiten. Zudem spielen Angehörige im Behandlungsablauf eine Vermittlerrolle. Für Behandelnde besteht das „Gegenüber“ im Versorgungsalltag also häufig aus 2 Personen (Dyade) [17, 18]. Wesentliche Einflussfaktoren der Behandelnden werden von Doherty [6] in ihrer Übersichtsarbeit aus überwiegend qualitativen Studien resümiert. Zu diesen Einflussfaktoren gehören das spezifische Fachwissen der Behandelnden über die Patientengruppe, ihre Fähigkeit, mit MmgB umzugehen, sowie ihre praktische Erfahrung. Die Gestaltung der Arzt-Patienten-Beziehung ist damit von besonderer Bedeutung. Dies betrifft eine Gesprächsführung, in die die MmgB und ihre Angehörigen aktiv einbezogen werden, aber auch wie mit Angst und Scham während einer Untersuchung umgegangen wird. Zudem ist eine hohe Behandlerkontinuität über einen längeren Zeitraum wichtig. Cantrell [5] ordnet die identifizierten Barrieren und förderlichen Faktoren aus Studien des Publikationszeitraums 2002–2018 in ein dreiphasiges Ablaufmodell ein. Dieses Modell zur Inanspruchnahme der medizinischen Versorgung durch MmgB ist gegliedert in:

- 1. das Erkennen eines Behandlungsbedarfs durch den MmgB oder Angehörige,
- 2. den Zugang zur Gesundheitsversorgung und
- 3. den eigentlichen Untersuchungsablauf.

In Deutschland ist sowohl der Zugang zum Gesundheitssystem [13, 19] als auch die Situation der ambulanten medizinischen Versorgung von MmgB wissenschaftlich weniger untersucht und erhobene Daten sind nur vereinzelt publiziert [20–22]. Die MmgB selbst kamen „bisher kaum zu Wort“ [23]. In einer Übersichtsarbeit aus dem Jahr 2014 kann Hasseler [8] auf keine Studiendaten aus Deutschland zurückgreifen. Dieses Defizit wird auch im TB-LMmB [24] festgestellt. Wetzels [25] Analyse der GEDA-Daten 2014/2015 zu Barrieren der Inanspruchnahme von MmgB ist auf die Wohnbevölkerung begrenzt und kann anhand des Indexmerkmals Grad der Behinderung den Kreis MmgB nicht von der größeren Zahl körperlich beeinträchtigter und behinderter abgrenzen. Auch andere offizielle Bevölkerungserhebungen beinhalten keine spezifischen Daten zu MmgB [26].

Die vorliegende Untersuchung erhebt daher Daten zu Barrieren und förderliche Faktoren in der ambulanten medizinischen Versorgung von MmgB in Deutschland. Sie berücksichtigt die Perspektive der MmgB sowie die ihrer begleitenden Personen, hier Angehörige genannt, und ergänzt sie um die Sicht ihrer Hausärzt:innen. Die thematische Gliederung und nachfolgende Darstellung folgt dem Ablaufmodell von Cantrell [5]. Diese Untersuchung ist Teil eines vom Landeszentrum Gesundheit Nordrhein-Westfalen geförderten Projektes zur Erfassung der medizinischen Versorgung von MmgB [27].

## Methoden

Die Querschnittstudie untersucht MmgB ab 18 Jahren, die in Werkstätten für behinderte Menschen (WfbM) arbeiten, da dort ein großer Teil der erwachsenen MmgB erreichbar ist [28]. Rekrutierungsorte sind 3 WfbM in 3 Städten Nordrhein-Westfalens, die in ihrer Größe, Gliederung und den Aufgaben typisch für WfbM in Deutschland sind und der Studiendurchführung zustimmten. Einschlusskriterien sind der Beschäftigtenstatus in der WfbM und ein Mindestalter von 18 Jahren. Ausschlusskriterium ist eine fehlende schriftliche Einverständniserklärung des MmgB oder der rechtlichen Vertretung zur Teilnahme. Die Studie erhebt Aspekte des Zugangs zur ambulanten medizinischen Versorgung von MmgB aus ihrer eigenen Perspektive, aus der ihrer Angehörigen, die sie bei Gesundheitsproblemen und Arztbesuchen unterstützen, und aus Perspektive ihrer Hausärzt:innen.

Ein validiertes Erhebungsinstrument existiert hierfür nicht. Literaturgestützt wurden 3 unterschiedliche Fragebögen entwickelt, einer für jede Perspektive [17, 29–32]. Der Fragebogen für MmgB und der für die Angehörigen erheben Daten zu Aspekten des Erkennens eines Behandlungsbedarfs, zum Zugang zur Gesundheitsversorgung und zum Untersuchungsablauf sowie zur Bewertung der Versorgung und Verbesserungsvorschläge. Zusätzlich wurden soziodemografische und gesundheitliche Daten erhoben. Der Fragebogen für MmgB entspricht grundsätzlich dem Fragebogen für Angehörige, ist aber in Leichter Sprache abgefasst und beinhaltet nicht die komplexer formulierten Fragen. Der Fragebogen für Hausärzt:innen erhebt Häufigkeit, Erfahrung und Aufwand bei der Untersuchung von MmgB sowie Ausstattung und Vorbereitung der eigenen Praxis auf deren Behandlung. Der Fragebogen für MmgB wird zusammen mit einer Interviewerin ausgefüllt, Angehörige und Hausärzt:innen füllen ihn selber aus. Die Fragebögen wurden mit Personen der 3 Gruppen auf Verständlichkeit vorgetestet.

Die Datenerhebung erfolgte von Februar bis Dezember 2016. In 2 WfbM fand initial eine Informationsveranstaltung für die MmgB und deren Angehörige statt. Alle 940 MmgB erhielten von den Werkstattmitarbeitenden ein Informationsschreiben, eine Einverständniserklärung und den Fragebogen für Angehörige. Nach Vorliegen der Einverständniserklärung führte eine Studienmitarbeiterin das Interview mit den MmgB in der WfbM durch. Die Hausärzt:innen erhielten ihren Fragebogen von der Person mit geistiger Behinderung bzw. einem Angehörigen und sandten ihn postalisch an die Studienmitarbeiterin zurück. Mit 2 Erinnerungsschreiben wurden die MmgB an die Studie erinnert und mit dem zweiten Schreiben wurden 10 Gutscheine im Wert von je 30 € in jeder WfbM als Teilnahmeanreiz verlost.

Zu allen Nicht-Teilnehmenden liegen auf Werkstattebene aggregierte und damit anonymisierte Angaben zu Alterskategorien sowie Geschlecht und für 2 WfbM auch zur Wohnform vor. Die Daten der Fragebögen wurden eingegeben, der Fehlerschätzer, ermittelt durch Zweiteingabe von 10 % der Daten, liegt bei 0,13 %. Die Datenauswertung mit dem Statistikprogramm SPSS Version 22 (IBM, Armonk, NY, USA) erfolgte überwiegend deskriptiv und zum Gruppenvergleich zwischen MmgB und deren Angehörigen mit dem T‑Test für verbundene Stichproben sowie zwischen Teilnehmenden und Nicht-Teilnehmenden mit dem Chi-Quadrat-Test. Das Signifikanzniveau lag explorativ bei *p* = 0,05, mit Bonferroni-Korrektur auf multiples Testen bei *p* < 0,001.

## Ergebnisse

### Stichprobe

Bei einer Teilnahmerate von 19,3 % aller 940 MmgB in 3 WfbM liegen auswertbar 176 Angehörigenbögen und 136 Fragebögen von MmgB vor. Für 133 Teilnehmende liegen sowohl Bögen der Angehörigen als auch der MmgB vor. Von 42 Teilnehmenden liegen Bögen ihrer Hausärzt:innen vor.

Die teilnehmenden MmgB sind im Mittel 40 Jahre alt (SD 13,8), zu 61,3 % männlich und 22,9 % weisen einen Migrationshintergrund auf (mindestens ein Elternteil oder sie selbst sind nicht in Deutschland geboren). 41,7 % wohnen bei Angehörigen, 30,3 % in Wohnheimen und 14,9 % im Betreuten Wohnen. Der Vergleich der verfügbaren Merkmale Alter, Geschlecht und Wohnform mit den Nicht-Teilnehmenden ist in der Tabelle A 1 im Onlinematerial aufgeführt und zeigt keine erheblichen Unterschiede.

Bei 75,0 % der Teilnehmenden besteht eine gesetzliche Betreuung. Die Laufmobilität ist bei 74,2 % nicht eingeschränkt, 14,1 % benötigen irgendeine Form der Gehhilfe. Den allgemeinen Gesundheitszustand schätzen die Angehörigen bei 16,8 % als weniger gut oder schlecht ein, bei 83,2 % als (sehr) gut oder ausgezeichnet. 68,2 % weisen einen Grad der Behinderung von 100 auf, 28,3 % von 50 bis 95. Die Angehörigen sind zu 63,9 % Familienangehörige und 34,3 % Mitarbeitende der Wohn- oder Werkstätten, ihr mittleres Alter beträgt 52 Jahre (SD 14,3), 75,7 % sind Frauen. Die Angaben zur Stichprobe sind in Tabelle A 1 und A 2 im Onlinematerial aufgeführt.

### Erkennen eines Behandlungsbedarfs

Aus Sicht aller Angehörigen teilen 60,7 % der MmgB ihre Beschwerden immer oder meistens den Angehörigen mit, bei 27,7 % müssen Angehörige Beschwerden selber erfassen, da die MmgB diese nie oder selten mitteilen (Tab. 1). MmgB geben mit 81,5 % signifikant häufiger als ihre Angehörigen (74,0 %) an, sich immer oder meistens mitzuteilen. Die Beschwerden teilen MmgB (Mehrfachnennung) bei 71,6 % aller Angehörigen in Worten, bei 1,1 % in Gebärdensprache mit. Doch auch Gesten und Blicke sind bei 38,6 % Ausdrucksmittel und 21,6 % vermitteln Beschwerden auch durch Körperreaktionen oder Lautieren. Alle MmgB, mit denen der Fragebogen ausgefüllt wurde, waren erwartungsgemäß in der Lage, Beschwerden verbal mitzuteilen.

**Table 1:** 

	Alle Angehörigen		Angehörige befragter Menschen mit geistiger Behinderung		Menschen mit geistiger Behinderung		*p*-Wert (T-Test)
	*N*	%	*N*	%	*N*	%	
*Mitteilen von Beschwerden an Angehörige* (*N* = 172; 131; 124)							
Nie	26	15,0	9	6,9	1	0,8	0,022
Selten	22	12,7	11	8,4	6	4,8	
Manchmal	19	11,0	14	10,7	16	12,9	
Meistens	35	20,2	32	24,4	13	10,5	
Immer	70	40,5	65	49,6	88	71,0	
*Mitteilungsart von Beschwerden an Angehörige;* Mehrfachantworten (*N* = 176; 133)							
Mit Worten	126	71,6	114	85,7	Nicht erhoben		
Mit Worten und Gesten	43	24,4	30	22,6			
Mit Gesten und Blicken	25	14,2	8	6,0			
Mit Gebärdensprache	2	1,1	0	0			
Durch Lautieren	12	6,8	0	0			
Durch Körperreaktionen	26	14,8	7	5,3			
Mit Hilfe von Tafeln	1	0,6	0	0			
Mit Hilfe eines Computers	3	1,7	0	0			
Handschriftlich	1	0,6	1	0,8			
Sonstiges	9	5,1	6	4,5			
*Arztbesuch aus eigenem Antrieb* (*N* = 168; 128; 126)							
Nie	84	49,1	50	39,1	67	53,2	0,621
Selten	14	8,2	12	9,4	6	4,8	
Manchmal	11	6,4	11	8,6	5	4,0	
Meistens	29	17,0	27	21,1	13	10,3	
Immer	30	17,5	28	21,9	35	27,8	
*Angst vor Arztbesuchen* (*N* = 174; 133; 132)							
Nie	75	42,6	61	45,9	83	62,9	0,553
Selten	37	21,0	29	21,8	20	15,2	
Manchmal	48	27,3	34	25,6	20	15,2	
Meistens	9	5,1	6	4,5	8	6,1	
Immer	5	2,8	3	2,3	1	0,8	
*Begleitung zum Arztbesuch* (*N* = 173; 132; 131)							
Selbstständig	30	17,3	29	22,0	34	26,0	0,485
Regelhaft mit Begleitperson	128	74,0	89	67,4	87	66,4	
Manchmal mit Begleitperson	13	7,5	13	9,8	9	6,9	
Der Arzt kommt zur ihr	2	1,2	1	0,8	1	0,8	
*Besonderheiten, die Angehörige vor Arztbesuchen beachten müssen;* Mehrfachantworten (*N* = 176; 133)							
Arzttermine überschneiden sich mit meiner Arbeitszeit	87	49,4	66	49,6	Nicht erhoben		
Die betreute Person akzeptiert keine Wartezeit in der Praxis	21	11,9	11	8,3			
Es wird eine zweite Begleitperson benötigt	9	5,1	4	3,0			
Sonstiges	17	9,7	9	6,8			
Keine Besonderheiten	78	44,3	60	45,1			
*Es ist zeitlich schwer, die von mir betreute Person zum Arzt zu begleiten* (*N* = 166; 124)							
Trifft zu	8	4,8	6	4,8	Nicht erhoben		
Trifft eher zu	17	10,2	14	11,3			
Trifft eher nicht zu	59	35,5	46	37,1			
Trifft nicht zu	82	49,4	58	46,8			
*Sind Empfehlungen zur Teilnahme an Krebsfrüherkennungsuntersuchungen bekannt?* (*N* = 173; 131)							
Ja	141	81,5	104	79,4	Nicht erhoben		
Nein	32	18,5	27	20,6			
*Ist das Angebot zum Gesundheits-Check-up 35 bekannt?* (*N* = 168; 125)							
Ja	129	76,8	93	74,4	Nicht erhoben		
Nein	39	23,2	32	25,6			
*Sind Impfempfehlungen gemäß Ständiger Impfkommission bekannt?* (*N* = 172; 130)							
Ja	135	78,5	102	78,5	Nicht erhoben		
Nein	37	21,5	28	21,5			
*Sind Angebote der Krankenkassen zu präventiven oder gesundheitsfördernden Maßnahmen bekannt (z.* *B. Sport‑, Ernährungs‑, Entspannungskursen, Gewichtsreduktion, Raucherentwöhnung)?* (*N* = 171; 128**)**							
Ja	103	60,2	77	60,1	Nicht erhoben		
Nein	68	39,8	51	39,8			
*Gründe für die Nicht-Teilnahme an präventiven oder gesundheitsfördernden Maßnahmen (z. B. Sport‑, Ernährungs‑, Entspannungskursen, Gewichtsreduktion, Raucherentwöhnung)*; Mehrfachantworten (*N* = 176; 133)							
Ich wusste nicht, dass solche Kurse angeboten werden	43	24,4	32	24,0	Nicht erhoben		
Kursinhalte sind nicht für Menschen mit geistiger Behinderung geeignet	41	23,3	27	20,3			
Die aufwändige Organisation (Transport zu den Kursen, Begleitung während des Kurses) muss durch die Angehörigen/Betreuer geleistet werden	32	18,2	20	15,0			
Die von mir betreute Person wollte nicht teilnehmen	29	16,5	28	21,1			
Die von mir betreute Person hat keinen Bedarf an diesen Kursen	29	16,5	23	17,3			
Die Kosten für den Präventionskurs sind zu hoch	7	4,0	4	3,0			
Ich konnte keine Betreuung/Begleitung für die Kursdauer organisieren	6	3,4	0	0			
Ich konnte keinen Transport zum Kurs organisieren	4	2,3	2	1,5			
Anderer Grund	8	4,5	4	3,0			
Weiß ich nicht	13	7,4	10	7,5			
*Gründe für Nicht-Teilnahme an zusätzlich benötigten Heilmittel-Therapien (z. B. Physiotherapie, Ergotherapie, Logopädie)*; Mehrfachantworten (*N* = 78; 58)							
Muss noch organisiert werden	24	30,8	21	36,2	Nicht erhoben		
Fehlende Kostenübernahme	24	30,8	14	24,1			
Betreute Person hat kein Interesse	18	23,1	15	25,9			
Fehlende Begleitperson	16	20,5	9	15,5			
Kein Überblick über das bestehende Angebot	15	19,2	9	15,5			
Mangel an Einrichtungen	9	11,5	4	6,9			
Wartezeiten	6	7,7	4	6,9			
Andere Gründe	12	15,4	9	15,5			

Eine Arztpraxis suchen 34,5 % der MmgB aller Angehörigen immer oder meistens aus eigenem Antrieb auf, bei Angehörigen der befragten MmgB sind dies 43 %. Angst vor Praxisbesuchen ist für alle Angehörigen zu 63,6 % nie oder selten ein Thema, die MmgB selbst geben dies sogar zu 78,1 % an, was deren Angehörige aber nur zu 67,7 % so sehen. Alle Angehörigen geben zu 74,0 % an, regelhaft gemeinsam mit den MmgB zu Praxen zu gehen, und zu 17,3 %, dass die MmgB selbstständig gehen. MmgB berichten zu 26,0 % selbstständig Praxen aufzusuchen und nur zu 66,4 % in Begleitung.

Bei der Terminvereinbarung geben 49,4 % aller Angehörigen das Abstimmen mit ihrer eigenen Arbeitszeit als häufigste organisatorische Besonderheit an, aber nur bei 15,0 % ist das Begleiten dann zeitlich eher schwierig oder schwierig. Die fehlende Wartetoleranz der MmgB in der Praxis ist für 11,9 % aller Angehörigen bei Arztbesuchen zu beachten. Für 44,3 % ist jedoch nichts Besonderes zu berücksichtigen.

Die Vorsorgemaßnahmen der gesetzlichen Krankenkassen zur Krebsfrüherkennung sind bei 81,5 % aller Angehörigen bekannt, zu Impfungen bei 78,5 % und zum Gesundheits-Check-up ab 35 Jahren bei 76,8 %. Präventive und gesundheitsförderliche Angebote sind mit 60,2 % weniger bekannt. Gründe daran, nicht teilzunehmen (Mehrfachnennung), sind für 24,4 %, die Angebote nicht zu kennen, und für 23,3 %, die Einschätzung, solche Angebote seien nicht für MmgB geeignet. Den Organisationsaufwand für eine Kursteilnahme nennen 18,2 % und mangelnde Teilnahmebereitschaft der MmgB 16,5 %. Gründe, an Heilmitteltherapien wie Physiotherapie, Ergotherapie oder Logopädie nicht teilzunehmen (Mehrfachnennung), die in einem bestimmten Umfang jährlich kostenfrei rezeptiert werden können, liegen zu 30,8 % am Organisieren der Therapien, aber bei 30,8 % auch an der fehlenden Kostenzusage der Krankenkasse, die bei einem erhöhten Bedarf notwendig ist. Weitere Gründe sind fehlendes Interesse der betreuten Person (23,1 %), eine fehlende Begleitperson (20,5 %) und ein fehlender Überblick über bestehende Angebote (19,2 %).

### Zugang zur Gesundheitsversorgung

Die meisten MmgB suchen regelmäßig 3 (28,7 %), 4 (17,8 %) oder 2 (15,5 %) Arztpraxen auf (Tab. 2). Schwierigkeiten, erfahrene Behandelnde zu finden, geben 70,1 % aller Angehörigen an. Diese haben zu 60,8 % keine und zu 25,1 % wenige Probleme, zur Praxis zu kommen. Bei 20,5 % gibt es unmittelbare Zugangsprobleme zu 1 Praxis und bei 10,6 % zu 2 oder mehr Praxen. Bei 30,9 % aller Angehörigen ist nie oder selten Informationsmaterial in Leichter Sprache in den Praxen vorhanden, jedoch können 47,3 % dies gar nicht beurteilen. 34,5 % haben bei ihrem letzten Praxisbesuch (Haus- oder Spezialfachärzt:innen) am gleichen Tag einen Termin bekommen, 23,7 % binnen einer Woche und 20,0 % binnen eines Monats. Für 12,7 % ist diese Wartezeit zu lang. 39,3 % aller Angehörigen geben keine oder höchstens 15 min Wartezeit für ihren letzten Praxisbesuch an, bei 22,8 % waren es mehr als 30 min.

**Table 2:** 

	Alle Angehörigen		Angehörige befragter Menschen mit geistiger Behinderung		Menschen mit geistiger Behinderung		*p*-Wert (T-Test)
	*N*	%	*N*	%	*N*	%	
*Anzahl regelmäßig aufgesuchter verschiedener Arztpraxen* (*N* = 174; 131)							
0	6	3,4	5	3,8	Nicht erhoben		
1	26	14,9	18	13,7			
2	27	15,5	20	15,3			
3	50	28,7	37	28,2			
4	31	17,8	24	18,3			
5	18	10,3	16	12,2			
6	10	5,7	7	5,3			
7	6	3,4	4	3,1			
*Schwierigkeiten, spezialisierte Ärzte zu finden* (*N* = 164; 122)							
Trifft zu	45	27,4	33	27,0	Nicht erhoben		
Trifft eher zu	70	42,7	53	43,4			
Trifft eher nicht zu	34	20,7	26	21,3			
Trifft nicht zu	15	9,1	10	8,2			
*Gibt es aufgrund der Behinderung Probleme, zum Arzt zu gelangen?* (*N* = 171; 131)							
Überhaupt keine	104	60,8	80	61,1	Nicht erhoben		
Wenige	43	25,1	36	27,5			
Einige	14	8,2	10	7,6			
Ziemlich viele	4	2,3	2	1,5			
Sehr viele	4	2,3	2	1,5			
Weiß ich nicht	2	1,2	1	0,8			
*Wenn ein Transport in Begleitung (durch einen speziellen Krankentransportdienst) nötig ist, ist es schwierig, einen solchen Transportdienst zu organisieren?* (*N* = 168; 124)							
Trifft zu	2	1,2	1	0,8	Nicht erhoben		
Trifft eher zu	9	5,4	4	3,2			
Trifft eher nicht zu	16	9,5	12	9,7			
Trifft nicht zu	20	11,9	14	11,3			
Diese Situation kam bisher nicht vor	89	53,0	71	57,3			
Weiß ich nicht	32	19,0	22	17,7			
*Praxen mit Zugangsproblemen* (z. B. kein Fahrstuhl vorhanden; *N* = 151; 113)							
0	104	68,9	75	66,4	Nicht erhoben		
1	31	20,5	25	22,1			
2	10	6,6	8	7,1			
3	5	3,3	4	3,5			
4	1	0,7	1	0,9			
*Hausarztpraxis für Rollstuhlfahrer erreichbar* (*N* = 167; 124)							
Ja	111	66,5	81	65,3	Nicht erhoben		
Nein	34	20,4	24	19,4			
Weiß ich nicht	22	13,2	19	15,3			
*Untersuchungsraum für Rollstuhlfahrer erreichbar* (*N* = 166; 123)							
Ja	106	63,9	77	62,6	Nicht erhoben		
Nein	20	12,0	13	10,6			
Weiß ich nicht	40	24,1	33	26,8			
*Sanitärbereich mit ausreichendem Platz für Rollstuhlfahrer* (*N* = 163; 120)							
Ja	31	19,0	27	22,5	Nicht erhoben		
Nein	43	26,4	26	21,7			
Weiß ich nicht	89	54,6	67	55,8			
*Schilder in der Arztpraxis gut lesbar* (*N* = 167; 124)							
Ja	106	63,5	79	63,7	Nicht erhoben		
Nein	17	10,2	12	9,7			
Weiß ich nicht	44	26,3	33	26,6			
*Informationsmaterial in Leichter Sprache ist in Arztpraxen vorhanden* (*N* = 165; 123)							
Immer	9	5,5	5	4,1	Nicht erhoben		
Meistens	11	6,7	9	7,3			
Manchmal	16	9,7	12	9,8			
Selten	18	10,9	12	9,8			
Nie	33	20,0	25	20,3			
Weiß ich nicht	78	47,3	60	48,8			
*Wie lang war die Zeit zwischen Terminvereinbarung und letztem Arztbesuch?* (*N* = 165; 123)							
Hat sofort einen Termin bekommen	57	34,5	37	30,1	Nicht erhoben		
Ein Tag	12	7,3	6	4,9			
2–6 Tage	27	16,4	22	17,9			
1 Woche bis 1 Monat	33	20,0	26	21,1			
Über 1 Monat	7	4,2	5	4,1			
Ist ohne Terminvereinbarung zum Arzt	13	7,9	13	10,6			
Termin bei diesem Anliegen nicht notwendig	7	4,2	6	4,9			
Kann mich nicht mehr erinnern/zu lange her	1	0,6	1	0,8			
Weiß ich nicht	8	4,8	7	5,7			
*Dauer zwischen Terminvereinbarung und Arztbesuch zu lang? *(*N* = 158; 117)							
Ja	20	12,7	16	13,7	Nicht erhoben		
Nein	130	82,3	93	79,5			
Weiß ich nicht	8	5,1	8	6,8			
*Ärzten steht genügend Zeit zur Verfügung* (*N* = 167; 125)							
Trifft zu	35	20,8	23	18,4	Nicht erhoben		
Trifft eher zu	45	26,8	36	28,8			
Trifft eher nicht zu	69	41,1	53	42,4			
Trifft nicht zu	18	10,7	13	10,4			
*Wartezeit in der Praxis beim letzten Arztbesuch* (*N* = 158; 119; 91)							
Keine Wartezeit	5	3,2	2	1,7	31	34,1	< 0,001
Bis 15 min	57	36,1	40	33,6	33	36,3	
Bis 30 min	60	38,0	48	40,3	18	19,8	
Bis 60 min	28	17,7	21	17,6	5	5,5	
Bis 2 h	8	5,1	8	6,7	4	4,4	
*War die Wartezeit in der Praxis für die von Ihnen betreute Person zu lang/war Ihnen die Wartezeit zu lang?* (*N* = 160; 123; 92)							
Nein	123	76,9	96	78,0	65	70,7	< 0,001
Ja	37	23,1	27	22,0	27	29,3	
*Konnte die von Ihnen betreute Person/konnten Sie in den letzten 12 Monaten einmal einen Arzttermin kurzfristig nicht einhalten?* (*N* = 166; 127; 96)							
Nein	136	81,9	105	82,7	69	71,9	< 0,001
Ja	30	18,1	22	17,3	27	28,1	
*Wenn Ja, warum konnte sie/konnten Sie den Termin nicht einhalten? *Mehrfachantworten (*N* = 30; 22; 27)							
Keine Zeit/andere Termine	11	36,7	10	45,5	8	29,6	0,161
Gesundheitsbedingt/ihr ging es zu schlecht	10	33,3	7	31,8	3	11,1	0,185
Es war keine Begleitperson vorhanden	8	26,7	6	27,3	5	18,5	0,083
Vergessen	5	16,7	5	22,7	4	14,8	0,327
Nicht mit der Arbeit vereinbar	2	6,7	2	9,1	0	0,0	–
Nicht mehr für notwendig erachtet	2	6,7	2	9,1	1	3,7	
Zu einem anderen Arzt gegangen	2	6,7	2	9,1	0	0,0	
Familiäre Gründe	1	3,3	1	4,5	0	0,0	
Arzt musste nach Hause kommen	1	3,3	1	4,5	1	3,7	
Angst vor Behandlung	1	3,3	1	4,5	0	0,0	
Kein Vertrauen zum Arzt	2	6,7	1	4,5	1	3,7	
Sonstiges	5	16,7	2	9,1	2	7,4	

MmgB erinnern signifikant häufiger eine kürzere Wartezeit als ihre Angehörigen. Als zu lang bewerten die Praxiswartezeit 23,1 % aller Angehörigen und mit 29,3 % signifikant mehr MmgB als deren Angehörige (22,0 %). Die verfügbare Zeit mit dem behandelnden Arzt beurteilt je die Hälfte der Angehörigen als (eher) genügend bzw. (eher) nicht genügend. In den letzten 12 Monaten konnten 18,1 % aller Angehörigen einen Arzttermin kurzfristig nicht einhalten. Die häufigsten Gründe (Mehrfachnennung) waren eine Terminkollision (36,7 %), eine schlechte gesundheitliche Verfassung des MmgB (33,3 %) und eine fehlende Begleitperson (26,7 %).

### Untersuchungsablauf in der Behandlungseinrichtung

79,3 % der MmgB sind mit ihren Ärzt:innen zufrieden und 51,5 % verstehen, was diese ihnen sagen, weitere 35,3 % zumindest teilweise (Tab. 3). Verstanden fühlen sich 72,8 %. Nach dem Arztbesuch geht es 71,3 % besser und 71,9 % halten sich an die Vereinbarungen.

**Table 3:** 

	Menschen mit geistiger Behinderung	
	*N*	%
*Sind Sie mit Ihren Ärzten zufrieden?* (*N* = 135)		
Ja	107	79,3
Nein	1	0,7
Teilweise	20	14,8
Weiß ich nicht	7	5,2
*Reden Ihre Ärzte so mit Ihnen, dass Sie verstehen, was die Ärzte Ihnen sagen?* (*N* = 136)		
Ja	70	51,5
Nein	9	6,6
Teilweise	48	35,3
Weiß ich nicht	9	6,6
*Fühlen Sie sich von Ihren Ärzten verstanden?* (*N* = 136)		
Ja	99	72,8
Nein	1	0,7
Teilweise	17	12,5
Weiß ich nicht	19	14,0
*Geht es Ihnen nach Arztterminen besser?* (*N* = 136)		
Ja	97	71,3
Nein	2	1,5
Teilweise	16	11,8
Weiß ich nicht	21	15,4
*Halten Sie sich an Vereinbarungen mit Ihren Ärzten?* (*N* = 135)		
Ja	97	71,9
Nein	1	0,7
Teilweise	19	14,1
Weiß ich nicht	18	13,3

Aus Sicht aller befragten Angehörigen treten Schwierigkeiten im Verlauf der Behandlung im Facharztgruppenvergleich am häufigsten bei Frauenärzt:innen (21,7 %), Zahnärzt:innen (18,9 %) und Augenärzt:innen (18,1 %; immer oder meistens) auf (Tab. 4). Die häufigsten Schwierigkeiten sind Verständigungsprobleme, Ängste und Unruhe der MmgB. Bei Frauenärzt:innen treten Ängste mit 20,0 % am häufigsten auf, 11,7 % der MmgB weigern sich, hier Untersuchungen durchführen zu lassen, auch Schmerzen (6,7 %) und fehlendes Vertrauen (8,3 %) werden hier als Schwierigkeiten benannt. Über alle Facharztgruppen werden 5–10 % der Untersuchungen, bei denen es Schwierigkeiten gibt, nicht durchgeführt.

**Table 4:** 

*Gibt es aufgrund der bestehenden Behinderung Schwierigkeiten bei der Durchführung diagnostischer und therapeutischer Maßnahmen?*												
**–**	**Allgemeinarzt**		**Neurologe**		**Frauenarzt**		**Augenarzt**		**Orthopäde**		**Zahnarzt**	
	*N* = 159	%	*N* = 90	%	*N* = 60	%	*N* = 121	%	*N* = 85	%	*N* = 148	%
Nie	93	58,5	53	58,9	30	50,0	64	52,9	55	64,7	78	52,7
Selten	28	17,6	15	16,7	8	13,3	16	13,2	11	12,9	19	12,8
Manchmal	26	16,4	12	13,3	8	13,3	15	12,4	10	11,8	21	14,2
Meistens	5	3,1	1	1,1	4	6,7	9	7,4	3	3,5	7	4,7
Immer	6	3,8	6	6,7	9	15,0	13	10,7	5	5,9	21	14,2
Weiß ich nicht	1	0,6	3	3,3	1	1,7	4	3,3	1	1,2	2	1,4
*Welche Schwierigkeiten treten bei der Durchführung diagnostischer und therapeutischer Maßnahmen auf?* Mehrfachantworten												
–	**Allgemeinarzt**		**Neurologe**		**Frauenarzt**		**Augenarzt**		**Orthopäde**		**Zahnarzt**	
	*N* = 159	%	*N* = 90	%	*N* = 60	%	*N* = 121	%	*N* = 85	%	*N* = 148	%
Ängste	17	10,7	5	5,6	12	20,0	9	7,4	3	3,5	29	19,6
Kommunikationsschwierigkeiten	42	26,4	22	24,4	12	20,0	24	19,8	12	14,1	26	17,6
Fehlendes Vertrauen	6	3,8	1	1,1	5	8,3	4	3,3	2	2,4	9	6,1
Mangelnde Einsichtsfähigkeit des Betroffenen	11	6,9	7	7,8	2	3,3	7	5,8	4	4,7	12	8,1
Schmerzen	7	4,4	0	0,0	4	6,7	0	0,0	3	3,5	8	5,4
Unruhe	21	13,2	16	17,8	8	13,3	15	12,4	8	9,4	21	14,2
Weigerung des Betroffenen	9	5,7	2	2,2	7	11,7	10	8,3	1	1,2	12	8,1
Andere Schwierigkeiten	6	3,8	3	3,3	4	6,7	9	7,4	4	4,7	5	3,4
*Finden die Untersuchungen auch statt, wenn es Schwierigkeiten bei der Durchführung gibt?*												
–	**Allgemeinarzt**		**Neurologe**		**Frauenarzt**		**Augenarzt**		**Orthopäde**		**Zahnarzt**	
	*N* = 73	%	*N* = 41	%	*N* = 31	%	*N* = 61	%	*N* = 34	%	*N* = 71	%
Nein	4	5,5	3	7,3	2	6,5	6	9,8	3	8,8	6	8,5
Selten	1	1,4	0	0,0	0	0,0	1	1,6	1	2,9	0	0,0
Ab und zu	2	2,7	2	4,9	2	6,5	3	4,9	2	5,9	5	7,0
Meistens	9	12,3	4	9,8	2	6,5	7	11,5	5	14,7	9	12,7
Ja, aber unter veränderten Voraussetzungen	21	28,8	10	24,4	14	45,2	16	26,2	10	29,4	18	25,4
Ja, immer	36	49,3	21	51,2	11	35,5	28	45,9	13	38,2	33	46,5
Weiß ich nicht	0	0,0	1	2,4	0	0,0	0	0,0	0	0,0	0	0,0
*Berät der Arzt die von Ihnen betreute Person ausführlich?*												
–	**Allgemeinarzt**		**Neurologe**		**Frauenarzt**		**Augenarzt**		**Orthopäde**		**Zahnarzt**	
	*N* = 156	%	*N* = 72	%	*N* = 56	%	*N* = 111	%	*N* = 69	%	*N* = 140	%
Gar nicht	3	1,9	2	2,8	1	1,8	2	1,8	2	2,9	2	1,4
Nicht ausreichend	4	2,6	2	2,8	0	0,0	3	2,7	1	1,4	0	0,0
Ja, kurz und knapp	16	10,3	8	11,1	9	16,1	18	16,2	14	20,3	17	12,1
Ja, ausführlich	61	39,1	24	33,3	23	41,1	40	36,0	22	31,9	54	38,6
Ja, sehr ausführlich	57	36,5	24	33,3	14	25,0	29	26,1	18	26,1	49	35,0
Beratung ausführlich, aber auch einfache Erklärungen versteht die betreute Person nicht	13	8,3	10	13,9	7	12,5	13	11,7	9	13,0	15	10,7
Weiß ich nicht	2	1,3	2	2,8	2	3,6	6	5,4	3	4,3	3	2,1
*Werden Sie als Begleitperson ausreichend durch den Arzt beraten?*												
–	**Allgemeinarzt**		**Neurologe**		**Frauenarzt**		**Augenarzt**		**Orthopäde**		**Zahnarzt**	
	*N* = 147	%	*N* = 73	%	*N* = 51	%	*N* = 100	%	*N* = 70	%	*N* = 128	%
Gar nicht	1	0,7	2	2,7	1	2,0	2	2,0	2	2,9	2	1,6
Nicht ausreichend	3	2,0	1	1,4	1	2,0	0	0,0	0	0,0	0	0,0
Ja, kurz und knapp	16	10,9	15	20,5	8	15,7	19	19,0	17	24,3	21	16,4
Ja, ausführlich	53	36,1	22	30,1	23	45,1	36	36,0	25	35,7	44	34,4
Ja, sehr ausführlich	73	49,7	33	45,2	16	31,4	40	40,0	24	34,3	60	46,9
Weiß ich nicht	1	0,7	0	0,0	2	3,9	3	3,0	2	2,9	1	0,8

Die Beratung des MmgB sehen alle Angehörigen am ausführlichsten bei Hausärzt:innen (75,6 %) und Zahnärzt:innen (73,6 %) und seltener bei Orthopäd:innen (58,0 %) und Augenärzt:innen (62,1 %). Diese Einschätzung ist in der Reihenfolge identisch mit ihrer eigenen Beratung durch die jeweilige Arztgruppe, allerdings liegen die Werte hier mit 85,8 % bei Hausärzt:innen und 70,0 % bei Orthopäd:innen zumeist um etwa 10 % höher.

*Beurteilung und Verbesserungsmöglichkeiten der medizinischen Versorgung* Insgesamt beurteilen 57,4 % aller Angehörigen die ambulante medizinische Versorgung als sehr gut oder völlig ausreichend (Tab. 5). Als besonders gut (Mehrfachnennung) beurteilen 52,8 % konkret die Terminabsprachen, 51,1 % die Offenheit im Umgang mit MmgB und 33,0 % sehen die Beratung als gut an. Die verfügbare Zeit der Ärzte, die Qualität der Diagnostik und die Wartezeit beurteilt nur noch etwa ein Viertel als besonders gut.

**Table 5:** 

	Alle Angehörigen	
	*N*	%
*Wie beurteilen Sie die ambulante medizinische Versorgung insgesamt?* (*N* = 169)		
Sehr gut	20	11,8
Völlig ausreichend	77	45,6
Mittelmäßig	68	40,2
Schlecht	4	2,4
*Welche Bereiche der ambulanten medizinischen Versorgung (ohne Krankenhaus und Zahnarzt) beurteilen Sie als besonders gut? *Mehrfachantworten (*N* = 176)		
Gute Terminabsprache	93	52,8
Offenheit im Umgang mit dem behinderten Menschen und dessen Bezugspersonen	90	51,1
Gute Information/Beratung	58	33,0
Arzt nimmt sich viel Zeit	52	29,5
Gute Diagnostik	46	26,1
Wenig Wartezeit	43	24,4
Nichts ist besonders gut	11	6,3
Andere Aspekte	2	1,1
*Aus welchen Gründen waren Sie in den letzten 12 Monaten unzufrieden mit der ambulanten medizinischen Versorgung (ohne Krankenhaus und Zahnarzt)?* Mehrfachantworten (*N* = 176)		
Wartezeit beim Arzt/im Wartezimmer	28	15,9
Zu wenig Zeit beim Arzt/im Arztzimmer/für Besprechung	23	13,1
Wurde nicht ernst genommen	19	10,8
Praxismitarbeiter unhöflich/respektlos	19	10,8
Wartezeit für Termin	18	10,2
Arzt unhöflich/respektlos	11	6,3
Behandlung/Behandlungsvorschläge entsprachen nicht Erwartung	10	5,7
Arzt macht keinen Hausbesuch	8	4,5
Falsche Behandlung/falsche Diagnose/Inkompetenz	4	2,3
Von mir gewünschte Behandlung wurde vom Arzt verweigert	3	1,7
Wegstrecke zur Praxis/keine Praxis in der Nähe	3	1,7
Abrechnung/Honorar/finanzielle Angelegenheiten	1	0,6
Sonstiges	5	2,8
Kein Grund zur Unzufriedenheit in den letzten 12 Monaten angegeben	121	68,8
*Waren Sie in den letzten 12 Monaten einmal so unzufrieden mit der ärztlichen Behandlung, dass Sie sich beschweren wollten?* (*N* = 171)		
Ja	28	16,4
Nein	143	83,6
*Wenn Ja, haben Sie sich dann tatsächlich beschwert?* (*N* = 27)		
Ja	7	25,9
Nein	20	74,1
*Gab es Ereignisse, in denen eine Erkrankung von ärztlicher Seite so spät festgestellt wurde, dass es dadurch zu lebensbedrohlichen Situationen kam?* (*N* = 170)		
Ja	14	8,2
Nein	149	87,6
Nicht einschätzbar	7	4,1
*Gab es eine dauerhafte Schädigung durch die späte Diagnosestellung? *(*N* = 14)		
Nein	7	50,0
Ja	6	42,9
Nicht einschätzbar	1	7,1

55 Personen (32,5 %) unter allen Angehörigen geben an, in den letzten 12 Monaten mit der ambulanten medizinischen Versorgung unzufrieden gewesen zu sein. Von allen 176 Angehörigen (Mehrfachnennung) sind 15,9 % mit der Praxiswartezeit unzufrieden, 13,1 % mit der Zeit für das Arztgespräch und 10,8 % fühlten sich nicht ernst genommen; ebenfalls 10,8 % erlebten Praxismitarbeitende als unhöflich oder respektlos, 10,2 % sind mit der Wartezeit bis zum Termin unzufrieden. 28 aller Angehörigen (16,4 %) wollten sich in den letzten 12 Monaten beschweren, was aber nur 7 auch taten.

61,5 % aller Angehörigen und auch 62,8 % der MmgB sowie 61,9 % deren Angehöriger wollen regelmäßige ärztliche Vorsorgeuntersuchungen lieber in der Praxis durchführen lassen und nur je etwa 20 % in der WfbM (Tab. 6). Eine aktive Erinnerung daran wünschen sich 74,7 % aller Angehörigen und zwar eher von ihren Ärzt:innen als von ihrer Krankenkasse oder beiden.

**Table 6:** 

	Angehörige (alle)		Angehörige befragter Menschen mit geistiger Behinderung		Menschen mit geistiger Behinderung		*p*-Wert (T-Test)
	*N*	%	*N*	%	*N*	%	
*Wo sollte eine regelmäßige ärztliche Vorsorgeuntersuchung (Gesundheits-Check-up) durchgeführt werden?* (*N* = 169; 126; 121)							
In der Werkstatt	35	20,7	24	19,1	25	20,7	0,226
In der Praxis	104	61,5	78	61,9	76	62,8	
Egal	30	17,8	24	19,1	20	16,5	
*Wünschen Sie sich eine Erinnerung an den Gesundheits-Check-up? *(*N* = 166; 125)							
Ja	124	74,7	98	78,4	Nicht erhoben		
Nein	37	22,3	24	19,2			
Weiß ich nicht	5	3,0	3	2,4			
*Erinnerung gewünscht durch:* (*N* = 118; 98)							
Arzt	67	56,8	56	57,1	Nicht erhoben		
Krankenkassen	42	35,6	33	33,7			
Arzt und Krankenkassen	9	7,6	9	9,2			
*Wo sollten Präventionskurse angeboten werden?* (*N* = 155; 115; 104)							
In der Werkstatt	103	66,4	74	64,3	53	51,0	0,099
Außerhalb der Werkstatt	30	19,4	25	21,7	36	34,6	
Egal	22	14,2	16	13,9	15	14,4	
*Mit wem würde die von Ihnen betreute Person/würden Sie den Kurs gerne besuchen?* (*N* = 131; 106; 87)							
Mit überwiegend anderen Menschen mit Behinderung	35	26,7	25	23,6	15	17,2	0,004
Mit überwiegend anderen Menschen ohne Behinderung	9	6,9	9	8,5	14	16,1	
Mit Menschen mit Behinderung und Menschen ohne Behinderung	87	66,4	72	67,9	58	66,7	
*Welche Möglichkeiten sehen Sie, die medizinische Versorgung von Menschen mit geistiger Behinderung zu verbessern?* Mehrfachantworten (*N* = 176; 133)							
Medizinische Versorgungszentren mit Ärzten, die auf Menschen mit geistiger Behinderung spezialisiert sind	105	59,7	75	56,4	Nicht erhoben		
Erstellung eines Verzeichnisses qualifizierter Ärzte und Therapeuten	91	51,7	68	51,1			
Änderung der Qualifikation des medizinischen Personals	62	35,2	44	33,1			
Integrierte Versorgung, bei der Krankenhausabteilungen mit entsprechender Fachkenntnis stärker als bisher für eine spezialisierte, ambulante Versorgung geöffnet werden	56	31,8	38	28,6			
Versorgungskomplexe, in denen Werkstätten, Tagesförderstätten, Kliniken, Wohnheime auf einem Gelände liegen, um dortiges Netzwerk zu nutzen	52	29,5	41	30,8			
Behandlung in sozialpädiatrischen Zentren (SPZ) über das 18. Lebensjahr hinaus, „Ausbau“ der SPZ	32	18,2	23	17,3			
Öffnung der Einrichtungen bzw. medizinische Möglichkeiten der Eingliederungshilfe	20	11,4	13	9,8			

Die WfbM ist für 66,4 % aller Angehörigen der bevorzugte Ort für Präventionskurse, doch nur für 51,0 % der MmgB selber bzw. 64,3 % deren Angehöriger. 34,6 % der MmgB möchten Präventionskurse außerhalb der WfbM besuchen. Zwei Drittel der Angehörigen und der MmgB wollen gemischte Kursgruppen für Menschen mit und ohne Behinderung, doch 16,1 % der MmgB möchten Kursgruppen überwiegend mit Menschen ohne geistige Behinderung, doppelt so viele wie in den Angehörigengruppen.

Möglichkeiten, die medizinische Versorgung der MmgB zu verbessern (Mehrfachnennung), sehen 59,7 % aller Angehörigen in medizinischen Zentren mit Behandelnden, die auf MmgB spezialisiert sind. 51,7 % befürworten die Erstellung eines Verzeichnisses mit speziell qualifizierten Behandelnden. Eine Änderung der Qualifikation des medizinischen Personals geben 35,2 % an. Eine Ansiedelung der ambulanten Versorgung an Krankenhäusern mit entsprechender Fachkenntnis ist für 31,8 % und verbundene Versorgungskomplexe aus medizinischer Versorgung, Wohnheim und WfbM für 29,5 % eine Verbesserungsmöglichkeit.

### Sicht der Hausärzt:innen

Hausärzt:innen geben zu 68,3 % an, wöchentlich oder täglich berufliche Kontakte zu MmgB zu haben (s. Onlinematerial, Tabelle A 3). Für 86,4 % liegt der Anteil dieser Patientengruppe an ihren Praxispatienten bei bis zu 5 %. Einrichtungen der Behindertenhilfe, wie beispielsweise Wohnheime, betreuen 35,7 % und zwar zu 46,7 % täglich oder wöchentlich. Den Mehraufwand für die Behandlung beziffern 52,6 % der Ärzt:innen mit bis zu einem Viertel, weitere 28,9 % mit bis zur Hälfte und 18,4 % als noch höher. 26,2 % haben sich spezifisch zum Thema weitergebildet.

92,7 % halten den Gesundheits-Check-up ab 35 Jahren und 72,5 % auch Präventionskurse für MmgB für sinnvoll (trifft zu und trifft eher zu), jedoch nur 48,8 % informieren die Patient:innen über Präventionskurse der Krankenkassen (s. Onlinematerial, Tabelle A 4). 70,7 % beschreiben ihre Praxisräumlichkeiten als barrierefrei und 53,6 % als technisch eingerichtet auf die besonderen Untersuchungsanforderungen. 63,5 % bieten Hausbesuche an. Informationsmaterial in Leichter Sprache gibt es in 7,3 % der Praxen. Für 57,5 % der Hausärzt:innen ist die Untersuchung von MmgB eine große zeitliche Belastung bzw. stellt diese für den Praxisablauf dar. In der Behandlungssituation mit MmgB fühlen sich 90,2 % sicher und 65,0 % verstehen Äußerungen und Anliegen der MmgB immer. 95,0 % sehen auch bei ihren Praxismitarbeitenden gute Kenntnisse im Umgang mit MmgB. Sprechstundenangebote in WfbM halten 35,0 % für eine mögliche Verbesserung. Bestehende Weiterbildungsangebote sind für 70,0 % nicht ausreichend, 55,0 % zeigen Interesse an Weiterbildung. Der erhöhte Versorgungsaufwand ist für 95,0 % in der Vergütung nicht adäquat berücksichtigt.

## Diskussion

Die vorliegende Erhebung zur ambulanten medizinischen und insbesondere zur (haus-)ärztlichen Versorgung von MmgB verdeutlicht die wichtige Vermittlerfunktion der Angehörigen und weist auf bestehende Barrieren hin, die sich in organisatorischen Abläufen und nicht zuletzt im erlebten Umgang zeigen. Als wichtige förderliche Hilfe sticht der Wunsch hervor, kompetente Behandelnde leichter finden oder sogar in Versorgungszentren erreichen zu können. Die Hausärzt:innen verdeutlichen den erhöhten, jedoch nicht vergüteten Behandlungsaufwand und geben spezifischen Fortbildungsbedarf an.

Wir möchten auch in der Diskussion dem Ablaufmodell nach Cantrell [5] folgen. Die medizinische Versorgung beginnt mit dem Erkennen eines Behandlungsbedarfs, den MmgB oft auch nonverbal mitteilen. Diesen zu erfassen und zu verstehen, ist die erste wichtige Aufgabe der Angehörigen bei der Unterstützung der MmgB in der Gesundheitsversorgung. Diese „Dyade“ aus MmgB und angehöriger Person ist für die Versorgung regelhaft von zentraler Bedeutung, da die Angehörigen eine wichtige Bewertungs- und Entscheidungsfunktion für den Eintritt ins Versorgungssystem übernehmen [1, 7, 16]. Die hohe Übereinstimmung der Angaben von MmgB und deren Angehörigen zeigt eine ähnliche Wahrnehmung der Versorgungssituation. Über die beschwerdebedingte Inanspruchnahme hinaus sind fast 80 % der Angehörigen über empfohlene präventive Bedarfsanlässe wie Krebsvorsorge, Impfung und Gesundheits-Check-up informiert und damit mehr als in der Allgemeinbevölkerung [27, 33]. Präventive und gesundheitsfördernde Angebote wie Sport- oder Ernährungskurse sind nur 60 % der Angehörigen bekannt, wobei bisher keine Daten vorliegen, wie gut MmgB durch Präventionsmaßnahmen erreicht werden [13].

Angehörige bahnen, organisieren und begleiten den Zugang ins Versorgungssystem und sind damit auch für den 2. Schritt im Versorgungsablauf nach Cantrell zentral. Insgesamt fällt es den meisten Angehörigen zeitlich nicht schwer, den Arztbesuch zu begleiten. Dies spricht für ihr erfahrenes und routiniertes Meistern der organisatorischen Abläufe. Der medizinische Versorgungsbedarf der MmgB ist durch das regelhafte Aufsuchen mehrerer Praxen gekennzeichnet. Hierbei sind räumliche Barrieren weniger eine Schwierigkeit, da die Mobilität selten eingeschränkt ist. Dies unterscheidet MmgB von Menschen mit körperlichen Beeinträchtigungen, für die häufiger räumlichen Zugangsbarrieren bestehen [13, 19]. Die Herausforderungen für MmgB sind eher Ängste vor dem Arztbesuch, für ihre Angehörigen eher die Abstimmung der Termine mit der eigenen Arbeitszeit und für beide die Wartezeit innerhalb der Praxis. Die mit der Wartezeit verbundene Erfahrung ist ein wichtiges Moment im Ablauf des Praxisbesuches, der öfter als kritisch angegeben wird. Als belastend erlebt wird hier einerseits die teils ängstliche Anspannung von MmgB vor Untersuchungen, andererseits aber auch die erlebte Sozialsituation im Wartebereich [6, 7, 16]. Ein Teil der Angehörigen gibt die Erfahrung an, in der Praxis nicht ernst genommen oder unhöflich bzw. respektlos behandelt worden zu sein. Zu beachten ist dabei, dass ein Drittel der Angehörigen in den letzten 12 Monaten kritische Erfahrungen in Praxen gemacht hat und 16 % so störende, dass eine Beschwerde in Betracht gezogen wurde. Dass nur ein kleiner Teil sich dann auch tatsächlich beschwert hat, verdeutlicht, wie schwer es fällt, solche Erfahrungen anzusprechen. Eine für Kritik offene Haltung in Praxen oder gar ein aktives Erfragen, was Patient:innen kritisch auffällt, könnte hilfreich sein, dies zu verbessern.

Auch im Untersuchungsablauf, dem 3. Schritt nach Cantrell, weisen die Ergebnisse auf besondere Anforderungen hin. Es kann der Eindruck bestehen, von seinen Ärzt:innen nicht richtig verstanden zu werden, deren Mitteilungen nicht richtig zu verstehen oder nicht ausführlich genug beraten zu werden, wobei Letzteres nur wenige Angehörige angeben. Es fällt auf, dass Praxen vorhandene Vermittlungshilfen in Form bebilderter oder in Leichter Sprache abgefasster Darstellungen selten einsetzen. Die Studie erhebt diesbezüglich nicht, ob diese Hilfen den Ärzt:innen unbekannt und unvertraut sind, sie ihre eigene Mitteilungsfähigkeit als geeigneter ansehen oder ob die Themen aus ihrer Sicht schneller mit den Angehörigen und über den MmgB hinweg besprochen werden können, was mögliche Gründe wären. Die Hausärzt:innen bestätigen die geringe Bedeutung solcher Informationshilfen in ihren Praxen und die meisten geben an, MmgB zu verstehen und sich im Umgang mit ihnen sicher zu fühlen. Von besonderer Bedeutung im Verlauf der Praxiskonsultation sind körperliche Untersuchungen. Bei MmgB äußern sich dabei Schwierigkeiten konkret vor allem in Form von Ängsten, Verständigungsproblemen und Unruhe. So können Untersuchungen teilweise nicht durchgeführt werden bzw. müssen angepasst werden, wobei sich diese Schwierigkeiten je nach medizinischem Fachgebiet unterscheiden. Im Hinblick auf den Arbeitsaufwand geben die Hausärzt:innen einen auch zeitlich höheren Behandlungsaufwand bei MmgB an, ohne dies adäquat vergütet zu bekommen [4].

Insgesamt zeigen die Ergebnisse, dass auch in deutschen Praxen die bekannten Schwierigkeiten im Versorgungsablauf bestehen, wie sie Cantrell in ihrer Übersicht zusammengestellt hat. Und es zeigt sich ebenso die hohe Bedeutung der von Doherty herausgearbeiteten Faktoren zum Einfluss der Behandelnden [6]. Zentral sind Fachkenntnisse über spezifische Gesundheitskonstellationen, verbunden mit praktischer Erfahrung im Umgang mit MmgB. Die Beziehungsgestaltung, sowohl im organisatorischen Teil des Praxisbesuches mit dem Team als auch in der Arzt-Patienten-Beziehung, verlangt Fähigkeiten im Umgang mit ungewohnten Mitteilungsformen, affektiven und körperlichen Reaktionen. Damit ist auch in den Gesundheitseinrichtungen eine annehmende und respektvolle Grundhaltung im Umgang mit MmgB im Sinne einer aktiven Inklusionsbereitschaft wichtig und zu fördern [34]. Bei den Hausärzt:innen besteht Fortbildungsinteresse zur gesundheitlichen Versorgung von MmgB, die Mehrzahl konstatiert aber auch ein nur geringes Angebot hierzu.

Ein grundlegendes Zugangshindernis besteht für die meisten Angehörigen darin, nur (eher) schwer Praxen zu finden, die auf MmgB eingestellt sind. Naheliegenderweise wünschen sich viele entsprechende Verzeichnisse. In den strukturellen Veränderungen, die Angehörige als Verbesserungsmöglichkeiten angeben, vermischen sich praktische Zugangsaspekte mit medizinischen und Inklusionsaspekten. Vorsorgeuntersuchungen wie den Gesundheits-Check-up auch in WfbM durchzuführen, stimmen nur 20 % zu, die Mehrheit zieht die Hausarztpraxis vor. Die meisten wünschen sich von ihren Behandelnden oder ihrer Krankenkasse an Vorsorgeuntersuchungen erinnert zu werden. Medizinische Versorgungszentren mit auf MmgB spezialisierten Behandelnden sieht über die Hälfte der Angehörigen positiv. Seit Ende der Erhebung gibt es hierzu auch eine neue Entwicklung. Das Fünfte Buch Sozialgesetzbuch (SGB V) ermöglicht nun medizinische Zentren für Erwachsene mit Behinderung (MZEB) einzurichten, wobei deren Etablierung bisher nur langsam voranschreitet [35] und zu beachten ist, dass sie nicht für die Grundversorgung konzipiert sind.

### Limitationen und Stärken

Trotz mehrfacher Erinnerung an die Studienteilnahme liegt die Teilnahmerate bei nur 19,3 %. Die Bereitschaft der Teilnehmenden könnte hier aufgrund eher positiver Erfahrungen mit der Versorgung größer und die Ergebnisse dadurch positiv verzerrt sein. Ebenso stellen auch die teilnehmenden Hausärzt:innen eher eine erfahrenere und engagiertere Auswahl dar, so wie heute wohl die Mehrzahl der MmgB über engagierte Ärzt:innen versorgt werden [36].

Erhebungsmethodisch kann bei den persönlich befragten MmgB das Antwortverhalten im Sinne sozialer Erwünschtheit und durch Interpretation der Antworten durch die Interviewerin verzerrt sein.

Die Beleuchtung der Aspekte des Zugangs zur ambulanten medizinischen Versorgung von MmgB aus 3 Perspektiven – MmgB, ihrer Angehörigen und ihrer Hausärzt:innen – stellt eine Stärke dieser Studie dar.

## Fazit und Ausblick

Bei der medizinischen Versorgung von Menschen mit geistiger Behinderung (MmgB) ist es besonders wichtig, dass sowohl die Bedürfnisse und Anforderungen der MmgB beachtet als auch deren Angehörige aktiv einbezogen werden. Dabei beinhalten Terminvereinbarung, Praxiswartezeiten und weitere organisatorische Anforderungen bereits wichtige Inklusionsaspekte. Auch sind medizinische Besonderheiten und eine gute Beziehung der Behandelnden zu den Patient:innen und Angehörigen während der Untersuchung von großer Bedeutung, wozu Fortbildungen gefördert werden sollten. Als praktische Hilfen sollten zum einen Listen mit Praxen erfahrener Behandelnder verfügbar sein. Zum anderen wäre eine wohnortnahe interdisziplinäre und interprofessionelle Versorgungsmöglichkeit und für spezielle Bedarfe auch der Ausbau erreichbarer Versorgungszentren (MZEB) wünschenswert.

Als Forschungsausblick legt die vorliegende Studie nahe, künftig vertiefend mit qualitativen Methoden die Interaktionen zwischen MmgB, Angehörigen und dem Praxisteam zu untersuchen, um problematische und förderliche Faktoren und Muster zu identifizieren. Darüber hinaus sind Versorgungsanforderungen und -barrieren für noch stärker hilfs- und pflegebedürftige MmgB zu erforschen, die nicht in WfbM tätig sind. Ein weiterer Anlass, qualitativ vertiefend zu forschen, ist, Gründe für den hohen Anteil nicht teilnehmender MmgB – 81 % in dieser Studie – zu verstehen.

## Supplementary Information




