# Supplementary material for: People with intellectual disabilities (ID) in outpatient medical care: barriers to access and treatment process
Source: Bundesgesundheitsblatt Gesundheitsforschung Gesundheitsschutz. 2023 Jan 16;66(2):184–98. [Article in German] doi: 10.1007/s00103-023-03655-x (PMC9892072; doi:10.1007/s00103-023-03655-x)
Supplement: Supplementary file 1 [file 103_2023_3655_MOESM1_ESM.docx]

**Onlinematerial Tabelle A 1**: Soziodemografische und gesundheitliche Merkmale der Teilnehmenden und Vergleich mit den Nicht-Teilnehmenden

|  | **Teilnehmende** | | **Nicht-Teilnehmende** | | **gesamt** | ***p*-Wert*** |
| --- | --- | --- | --- | --- | --- | --- |
|  | **N** | **%** | **N** | **%** | **N** |  |
| **Institution** | | | | | | |
| Werkstatt 1 | 53 | 18,5 | 234 | 81,5 | 287 | 0,001 |
| Werkstatt 2 | 68 | 26,9 | 185 | 73,1 | 253 |  |
| Werkstatt 3 | 60 | 15,0 | 340 | 85,0 | 400 |  |
| gesamt | 181 | 19,3 | 759 | 80,7 | 940 | - |
| **Geschlecht** | | | | | | |
| männlich | 111 | 61,3 | 458 | 60,3 | 569 | 0,866 |
| weiblich | 70 | 38,7 | 301 | 39,7 | 371 |  |
| gesamt | 181 | 100 | 759 | 100 | 940 | - |
| **Alter** | | | | | | |
| <25 Jahre | 30 | 16,6 | 87 | 11,5 | 117 | 0,219 |
| 25-34 Jahre | 41 | 22,7 | 202 | 26,6 | 243 |  |
| 35-44 Jahre | 33 | 18,2 | 153 | 20,2 | 186 |  |
| 45-55 Jahre | 48 | 26,5 | 221 | 29,1 | 269 |  |
| >55 Jahre | 29 | 16,0 | 96 | 12,6 | 125 |  |
| gesamt | 181 | 100 | 759 | 100 | 940 | - |
| **Wohnform, Vergleichsdaten für Werkstatt 1 und 2** | | | | | | |
| allein | 8 | 6,7 | 30 | 7,2 | 38 | 0,045 |
| bei Verwandten | 44 | 37,0 | 179 | 42,8 | 223 |  |
| im Wohnheim | 41 | 34,5 | 99 | 23,7 | 140 |  |
| Betreutes Wohnen | 19 | 16,0 | 98 | 23,4 | 117 |  |
| in einer Wohngemeinschaft | 4 | 3,4 | 4 | 1,0 | 8 |  |
| mit dem Partner | 3 | 2,5 | 8 | 1,9 | 11 |  |
| gesamt | 119 | 100,1 | 418 | 100 | 537 | - |
| **Wohnform** (N = 175) | | | | | | |
| allein | 14 | 8,0 | nicht erhoben | | | |
| bei Verwandten | 73 | 41,7 |  |  |  |  |
| im Wohnheim | 53 | 30,3 |  |  |  |  |
| Betreutes Wohnen | 26 | 14,9 |  |  |  |  |
| in einer Wohngemeinschaft | 5 | 2,9 |  |  |  |  |
| mit dem Partner | 4 | 2,3 |  |  |  |  |
| **Migrationshintergrund** (N = 175) | | | | | | |
| ohne | 135 | 77,1 | nicht erhoben | | | |
| mit | 40 | 22,9 |  |  |  |  |
| **gesetzlicher Betreuer** (N = 176) | | | | | | |
| ja | 132 | 75,0 | nicht erhoben | | | |
| nein | 44 | 25,0 |  |  |  |  |
| **Mobilität der betreuten Person** (N = 163) | | | | | | |
| freies Laufen problemlos | 121 | 74,2 | nicht erhoben | | | |
| freies Laufen mit Mühe | 19 | 11,7 |  |  |  |  |
| Gehstock | 2 | 1,2 |  |  |  |  |
| Rollator | 6 | 3,7 |  |  |  |  |
| Rollstuhl | 11 | 6,7 |  |  |  |  |
| sonstige Unterstützung | 4 | 2,5 |  |  |  |  |
| **Gesundheitszustand** (N = 167) aus Angehörigenperspektive | | | | | | |
| schlecht | 4 | 2,4 | nicht erhoben | | | |
| weniger gut | 24 | 14,4 |  |  |  |  |
| gut | 105 | 62,9 |  |  |  |  |
| sehr gut | 28 | 16,8 |  |  |  |  |
| ausgezeichnet | 6 | 3,6 |  |  |  |  |
| **Gesundheitszustand** (N = 133) aus Perspektive Menschen mit geistiger Behinderung | | | | | | |
| schlecht | 6 | 4,5 | nicht erhoben | | | |
| weniger gut | 18 | 13,5 |  |  |  |  |
| gut | 75 | 56,4 |  |  |  |  |
| sehr gut | 23 | 17,3 |  |  |  |  |
| ausgezeichnet | 11 | 8,3 |  |  |  |  |
| **Grad der Behinderung** (N = 173) | | | | | | |
| kein Schwerbehindertenausweis | 3 | 1,7 | nicht erhoben | | | |
| unter 50 | 1 | 0,6 |  |  |  |  |
| 50-70 | 17 | 9,8 |  |  |  |  |
| 75-95 | 32 | 18,5 |  |  |  |  |
| 100 | 118 | 68,2 |  |  |  |  |
| weiß ich nicht | 2 | 1,2 |  |  |  |  |

* chi^2^-Test

**Onlinematerial Tabelle A 2**: Soziodemografische Angaben zu allen Angehörigen

|  | **N** | **%** |
| --- | --- | --- |
| **Geschlecht** (N = 169) | | |
| weiblich | 128 | 75,7 |
| männlich | 41 | 24,3 |
| **Alter** (N = 164) | | |
| <30 Jahre | 17 | 10,4 |
| 30-39 Jahre | 16 | 9,8 |
| 40-49 Jahre | 29 | 17,7 |
| 50-59 Jahre | 57 | 34,8 |
| 60-69 Jahre | 26 | 15,9 |
| 70-79 Jahre | 17 | 10,4 |
| >=80 Jahre | 2 | 1,2 |
| **Beziehung zum Menschen mit geistiger Behinderung** N = 169) | | |
| Angehörige | 108 | 63,9 |
| Mitarbeiter der Wohnstätten und Werkstätten | 58 | 34,3 |
| gesetzlicher Betreuer | 3 | 1,8 |

**Onlinematerial Tabelle A 3:** Patient:innen mit geistiger Behinderung in den Praxen der Hausärzt:innen

|  | **teilnehmende Ärzt:innen** | |
| --- | --- | --- |
|  | **N** | **%** |
| **Häufigkeit beruflicher Kontakte zu Menschen mit geistiger Behinderung** (N = 41) | | |
| seltener | 4 | 9,8 |
| monatlich | 9 | 22,0 |
| wöchentlich | 15 | 36,6 |
| täglich | 13 | 31,7 |
| **Wie hoch ist der prozentuale Anteil an Menschen mit geistiger Behinderung unter Ihren Patienten?** (N = 37) | | |
| 0-1 % | 14 | 37,8 |
| 2-3% | 8 | 21,6 |
| 4-5% | 10 | 27,0 |
| 6-10% | 4 | 10,8 |
| 11-15% | 1 | 2,7 |
| **Betreuen Sie Einrichtungen der Behindertenhilfe?** (N = 42) | | |
| ja | 15 | 35,7 |
| nein | 27 | 64,3 |
| **Wie häufig betreuen Sie die Einrichtungen der Behindertenhilfe?** (N = 15) | | |
| täglich | 1 | 6,7 |
| wöchentlich | 6 | 40,0 |
| monatlich | 7 | 46,7 |
| selten | 1 | 6,7 |
| **Bitte beziffern Sie den prozentualen Mehraufwand bei der Behandlung von Menschen mit geistiger Behinderung** (N = 38) | | |
| < 26% | 20 | 52,6 |
| 26-50% | 11 | 28,9 |
| 51-75% | 1 | 2,6 |
| 76-100% | 4 | 10,5 |
| > 100% | 2 | 5,3 |
| **Haben Sie sich in Bezug auf das Thema "gesundheitliche Versorgung von Menschen mit geistiger Behinderung" weitergebildet?** (N = 42) | | |
| ja | 11 | 26,2 |
| nein | 31 | 73,8 |

**Tabelle A 4:** Medizinische Versorgung von Menschen mit geistiger Behinderung aus Sicht der Hausärzt:innen

|  | | trifft zu | trifft eher zu | trifft eher nicht zu | trifft nicht zu |
| --- | --- | --- | --- | --- | --- |
| **Präventionsangebote** | | | | | |
| Den Gesundheits-Check-up für Menschen mit geistiger Behinderung halte ich für sinnvoll. | N = 41 | 27 | 11 | 3 | 0 |
|  | % | 65,9 | 26,8 | 7,3 | 0,0 |
| Ich glaube, dass Präventionskurse für Menschen mit geistiger Behinderung von Nutzen sind. | N = 40 | 17 | 12 | 10 | 1 |
|  | % | 42,5 | 30,0 | 25,0 | 2,5 |
| Ich informiere meine Patienten mit geistiger Behinderung über Präventionskurse, die von den Krankenkassen angeboten werden. | N = 41 | 5 | 15 | 13 | 8 |
|  | % | 12,2 | 36,6 | 31,7 | 19,5 |
| **Zugang zur Praxis** | | | | | |
| Meine Praxis/mein Arbeitsplatz ist "barrierefrei" im Hinblick auf die räumlichen Gegebenheiten. | N = 41 | 24 | 5 | 6 | 6 |
|  | % | 58,5 | 12,2 | 14,6 | 14,6 |
| Meine Praxis /mein Arbeitsplatz ist technisch auf die besonderen Anforderungen in der Behandlung von Menschen mit einer geistigen Behinderung eingerichtet. | N = 41 | 6 | 16 | 14 | 5 |
|  | % | 14,6 | 39,0 | 34,1 | 12,2 |
| In meiner Praxis/an meinem Arbeitsplatz gibt es verständliches medizinisches Informationsmaterial für Patienten mit geistiger Behinderung. | N = 41 | 0 | 3 | 23 | 15 |
|  | % | 0,0 | 7,3 | 56,1 | 36,6 |
| Ich biete Menschen mit geistiger Behinderung Hausbesuche an. | N = 41 | 17 | 9 | 11 | 4 |
|  | % | 41,5 | 22,0 | 26,8 | 9,8 |
| Die Untersuchung von Menschen mit geistiger Behinderung empfinde ich als eine große zeitliche Belas-tung/stellt eine zeitliche Belastung im Praxisablauf dar. | N = 40 | 5 | 18 | 14 | 3 |
|  | % | 12,5 | 45,0 | 35,0 | 7,5 |
| **Untersuchungsablauf in der Praxis** | | | | | |
| Bei der Behandlung von Menschen mit geistiger Behinderung fühle ich mich sicher. | N = 41 | 18 | 19 | 4 | 0 |
|  | % | 43,9 | 46,3 | 9,8 | 0,0 |
| Ich verstehe die Äußerungen bzw. das Anliegen eines Patienten mit geistiger Behinderung immer. | N = 40 | 9 | 17 | 11 | 3 |
|  | % | 22,5 | 42,5 | 27,5 | 7,5 |
| Ich habe das Gefühl, dass meine Arzthelfer/innen (MFA) sich gut mit dem Umgang von Menschen mit geistiger Behinderung auskennen. | N = 40 | 12 | 26 | 1 | 1 |
|  | % | 30,0 | 65,0 | 2,5 | 2,5 |
| **Verbesserungsmöglichkeiten der medizinischen Versorgung** | | | | | |
| Ich befürworte eine Sprechstunde in der Werkstatt für Menschen mit geistiger Behinderung. | N = 40 | 5 | 9 | 22 | 4 |
|  | % | 12,5 | 22,5 | 55,0 | 10,0 |
| Ich interessiere mich für Fort- und Weiterbildungs-angebote zum Thema "gesundheitliche Versorgung von Menschen mit einer geistigen Behinderung" | N = 40 | 12 | 10 | 12 | 6 |
|  | % | 30,0 | 25,0 | 30,0 | 15,0 |
| Das Weiterbildungsangebot zum Thema "gesundheitliche Versorgung von Menschen mit einer geistigen Behinderung" ist ausreichend. | N = 40 | 1 | 11 | 18 | 10 |
|  | % | 2,5 | 27,5 | 45,0 | 25,0 |
| Die Gebührenordnung/der einheitliche Bewertungsmaßstab ermöglicht keine adäquate Abrechnung des bei der Behandlung von Patienten mit geistiger Behinderung entstehenden Mehraufwands. | N = 40 | 26 | 12 | 1 | 1 |
|  | % | 65,0 | 30,0 | 2,5 | 2,5 |
